# Use of Bone Resorption Inhibitors in Metastatic Castration-Resistant Prostate Cancer—20 Years Later, and the Answer Is Still Yes

**DOI:** 10.1001/jamanetworkopen.2021.17159

**Published:** 2021-07-01

**Authors:** Samuel U. Takvorian, Naomi B. Haas

**Affiliations:** Division of Hematology and Oncology, Perelman School of Medicine, University of Pennsylvania, Philadelphia.

Prostate cancer is the most common type of cancer and the second leading cause of cancer death among men in the US. It preferentially metastasizes to bone, with up to 90% of men with metastatic disease having bone metastases. The presence and volume of bone metastases are associated with worse prognosis and the potential for skeletal complications, such as pathologic fractures, spinal cord compression, or the need for surgery or radiotherapy to bone. These skeletal-related events (SREs) collectively represent a clinically meaningful outcome that is often measured in clinical trials. Up to one-half of men with metastatic castration-resistant prostate cancer (mCRPC), the advanced and often fatal stage of disease, experience SREs, which are associated with considerable morbidity, decreased survival, and increased health care utilization and costs. Therefore, understanding strategies to optimize bone health and prevent skeletal complications remains a critical area of ongoing research and implementing them a component of high-quality prostate cancer care.

Over the past 20 years, 2 bone resorption inhibitors (BRIs) have emerged as alternatives for the prevention of skeletal complications among men with mCRPC: zoledronic acid (an intravenous bisphosphonate) and denosumab (a subcutaneous monoclonal antibody against the receptor activator of nuclear factor κB ligand [RANKL]). These agents differ mechanistically, with zoledronic acid preferentially inhibiting osteoclast proliferation and denosumab inhibiting an important factor in osteoclast maturation. In a placebo-controlled study of 643 men, zoledronic acid decreased the risk of SREs by 36% and delayed the time to first SRE by 167 days.^1^ In a subsequent phase 3 study of 1904 men, denosumab was superior to zoledronic acid, delaying the time to first SRE by 3.6 months (hazard ratio [HR], 0.82; 95% CI, 0.71-0.95).^2^ Zoledronic acid was more often associated with acute phase reactions and required monitoring of kidney function; denosumab conferred a higher risk of hypocalcemia. Rates of osteonecrosis of the jaw were comparably low. International guidelines endorse the use of either agent for the treatment of men with mCRPC, although neither agent has independently been associated with an overall survival benefit in a randomized study, and some argue that the marginal benefit of denosumab must be weighed against its dramatically higher cost (the annual cost of zoledronic acid is approximately $140 vs $29 000 for denosumab).^3,4^ Moreover, these pivotal studies were conducted before contemporary drug approvals, including those of abiraterone acetate, enzalutamide, radium-223, and cabazitaxel, each of which has been associated with extended survival and reduction in the risk of SREs. Thus, the benefits of zoledronic acid and denosumab in combination with contemporary drugs for the treatment of mCRPC remain incompletely understood.

Francini et al^5^ report the results of an international, multicenter, retrospective cohort study of men with mCRPC who initiated abiraterone acetate with prednisone as first-line therapy with or without the addition of a BRI (zoledronic acid or denosumab). Among 745 men with a median follow-up of almost 2 years, the authors found an overall survival benefit associated with the addition of a BRI (zoledronic acid or denosumab) to abiraterone with prednisone therapy (31.8 months with a BRI vs 23.0 months without a BRI; HR, 0.65; 95% CI, 0.54-0.79; *P* < .001), a benefit that persisted with multivariable adjustment and was most pronounced among men with high-volume disease.^5^

How should we interpret the significance of these findings? First, this study highlights the importance of bone-targeted therapy in current practice for men with mCRPC and bone metastases. Although data from a randomized study are lacking, multiple retrospective studies and post hoc analyses of phase 3 studies have suggested that the addition of a BRI to contemporary therapies for men with mCRPC may prolong survival in addition to preventing skeletal complications.^6,7^ Moreover, this study supports the hypothesis that BRIs may be particularly important for men with high-volume metastases,^7^ a hypothesis that warrants prospective testing in future studies.

Second, the study by Francini et al^5^ exposes real-world practice patterns across 8 major hospital systems in the US, Europe, and Canada. In a study population encompassing men with mCRPC and bone metastases for whom bone-targeted therapy is endorsed by international guidelines, more than 70% of participants were prescribed abiraterone acetate with prednisone alone and thus did not receive an indicated BRI.^5^ This gap is particularly noteworthy given the study's main finding of improved overall survival with concomitant bone-targeted therapy and highlights the need for implementation work (such as clinical pathways and behavioral nudges to promote adoption^4^) to bring evidence-based therapies to the patients who need them. Moreover, the authors found no association between the specific bone agent used (zoledronic acid vs denosumab) and overall survival.^5^ Although this finding must be considered preliminary given the limitations of a retrospective study, it adds to data suggesting that these agents are comparably beneficial; thus, decisions between them should focus on clinical factors, such as kidney function, patient preference, and cost.

As the authors admit, an important limitation of their study is its underrepresentation of patients from racial and ethnic minority groups, who often present with particularly aggressive prostate cancers and stand to benefit from these therapies. Although this limitation may, to some extent, reflect the geographic catchments of the institutions involved in the study, underrepresentation of racial and ethnic minority groups is endemic in cancer clinical trials, and the pivotal clinical trials leading to regulatory approvals of zoledronic acid and denosumab are no exception. A renewed focus on equitable participation in clinical research is needed.

Francini et al^5^ should be commended for assembling a large international contemporary cohort of men with mCRPC to provide insight into the important challenge of optimizing bone health in this population. Perhaps what we learned most from this study is that most of the time, men are not receiving guideline-concordant bone-targeted care. Should they be? The answer is still yes.
